# Residual Stress Homogenization of Hybrid Implants

**DOI:** 10.3390/bioengineering11111149

**Published:** 2024-11-15

**Authors:** Marta Sanjuán Álvarez, Daniel Robles, Javier Gil Mur, Saray Fernández-Hernández, Esteban Pérez-Pevida, Aritza Brizuela-Velasco

**Affiliations:** 1 Bioengineering Institute of Technology, International University of Catalonia, C/de la Inmaculada 22, Sarrià-Sant Gervasi, 08017 Barcelona, Spain; xavier.gil@uic.cat; 2 DENS-ia Research Group, Faculty of Health Sciences, Miguel de Cervantes European University, C/del Padre Julio Chevalier 2, 47012 Valladolid, Spainsfernandez@uemc.es (S.F.-H.); abrizuela@uemc.es (A.B.-V.)

**Keywords:** hybrid implants, residual stress, annealing heat treatment, microhardness, corrosion, mechanical behavior, finite element analysis (FEA)

## Abstract

Objectives: Hybrid implants commonly exhibit decreased corrosion resistance and fatigue due to differences in compressive residual stresses between the smooth and rough surfaces. The main objective of this study was to investigate the influence of an annealing heat treatment to reduce the residual stresses in hybrid implants. Methodology: Commercially pure titanium (CpTi) bars were heat-treated at 800 °C and different annealing times. Optical microscopy was used to analyze the resulting grain growth kinetics. Diffractometry was used to measure residual stress after heat treatment, corrosion resistance by open circuit potential (E_OCP_), corrosion potentials (E_CORR_), and corrosion currents (I_CORR_) of heat-treated samples, as well as fatigue behavior by creep testing. The von Mises distribution and the resulting microstrains in heat-treated hybrid implants and in cortical and trabecular bone were assessed by finite element analysis. The results of treated hybrid implants were compared to those of untreated hybrid implants and hybrid implants with a rough surface (shot-blasted). Results: The proposed heat treatment (800 °C for 30 min, followed by quenching in water at 20 °C) could successfully homogenize the residual stress difference between the two surfaces of the hybrid implant (−20.2 MPa). It provides better fatigue behavior and corrosion resistance (*p* ˂ 0.05, ANOVA). Stress distribution was significantly improved in the trabecular bone. Heat-treated hybrid implants performed worse than implants with a rough surface. Clinical significance: Annealing heat treatment can be used to improve the mechanical properties and corrosion resistance of hybrid surface implants by homogenizing residual stresses.

## 1. Introduction

Osseointegrated implants are known for their high survival rates (10-year survival of over 95%) but they are not complication-free [1,2]. Given their increasing popularity, implant complications are likely to become progressively more prevalent [3]. Hybrid implants were developed with the clinical goal of maintaining long-term peri-implant soft tissue health. As these implants have a machined coronal portion (4–5 mm), even when bone loss occurs and the first threads of the implants are exposed, they are less prone to biofilm formation and the growth of pathogenic flora, leading to peri-implant mucositis (inflammation of the soft tissue around the implant) or peri-implantitis (inflammation of the tissues together with irreversible bone loss) [4,5,6,7]. However, hybrid implants exhibit worse osseointegration, with lower reverse torque and bone-to-implant contact (BIC). Some researchers have advised against their use in the presence of unfavorable bone quality and quantity [8,9,10]. How the mechanical behavior of a hybrid implant is affected by having two contrasting surfaces (a smooth machined coronal surface and a rough sandblasted, large-grit, acid-etched (SLA) apical surface) remains uncertain. Furthermore, cyclic fatigue is closely related to the implant’s design and topography (e.g., surface roughness, wettability, residual stress, and chemical composition), and loading conditions [10,11,12,13].

Previous in vitro studies have investigated the mechanical properties of the three types of implant surfaces, namely machined, rough (with shot-blasting treatment with alumina grit), and hybrid [14,15,16]. These studies found that rough surfaces have a compressive residual stress that improves their fatigue strength (−200 MPa), due to the compressive stress of the impact caused by the blasting of the 220 µm alumina grit at a pressure of 5 bar on the titanium surface. The machined portion, however, has a residual stress of −20.2 MPa. This difference causes a higher corrosive potential at the interface of the hybrid implants, resulting in chemical degradation, which in turn, leads to corrosion pits that concentrate the stress [14] and worsen the fatigue relative to the rough and machined implants (Robles et al. p.10, parr.2, l.10-16). A finite element analysis found that hybrid implants had a minimal von Mises stress distribution in their machined coronal portion, particularly at the level of the cortical bone (Sanjuán et al. p.17, parr.7,l.60-64) [16]. Furthermore, corroborating available evidence suggests that hybrid implants exhibit worse mechanical behavior, with lower bending strength and worse fatigue behavior compared to rough and machined implants.

A surface treatment may improve the mechanical behavior of hybrid implants while maintaining their biological advantages. In the field of metallurgy, annealing heat treatment is commonly used to eliminate defects in metal structures by diffusion, thereby reducing the residual stresses and internal energy of the metal, increasing wettability and improving corrosion resistance. However, this treatment usually increases the number and size of crystals in the metal structure, thereby decreasing hardness and mechanical resistance [17,18].

The present in vitro experimental study aimed to explore the use of an annealing heat treatment to homogenize the different residual stresses in hybrid implants without extensively modifying their mechanical behavior and crystal grain structure [19]. The null hypothesis of this in vitro study is that annealing heat treatment of the mixed surfaces of hybrid implants does not improve the mechanical properties nor the corrosion resistance of the implants.

## 2. Material and Methods

### 2.1. Sample Preparation

**Figure 1:**
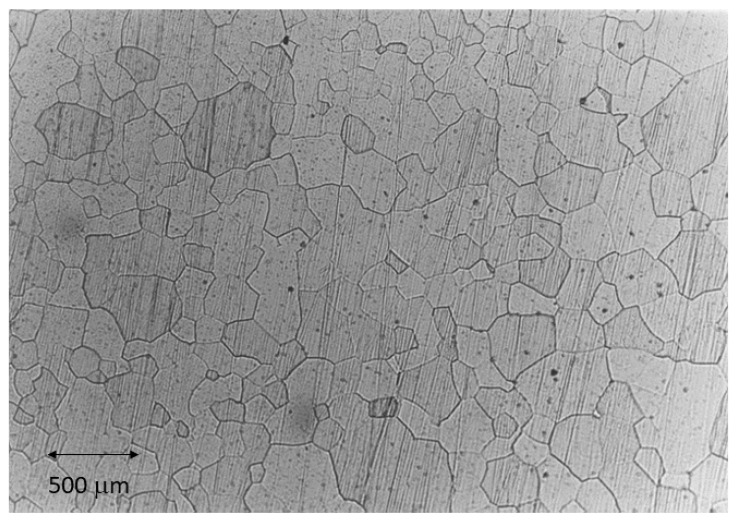
Microstructure of the α-phase of titanium, obtained by optical microscopy.

The SLA-treated commercially pure titanium used in the study was donated by SOADCO (Escaldes Engordany, Andorra) in the form of cylindrical bars. Forty disks measuring 3 mm in height and 5 mm in diameter were prepared. The chemical impurities of this alloy are indicated in Table 1, and Figure 1 shows that the microstructure of the material consisted of equiaxed alpha phase grains.

The titanium disks were sandblasted. Sandblasting involved bombarding the surface with alumina particles (average particle size = 220 μm), projected from a distance of 150 mm using a titanium spray gun under pressure (2.5 bar). This sandblasting treatment is commonly used for surface modification of dental implants in the clinical setting.

### 2.2. Annealing Heat Treatment

A temperature lower than the β-transus temperature (960 °C) is required to homogenize the residual stresses. Considering this and the temperature used for titanium heat treatment in other studies, 800 °C was adopted for this present study [19,20,21].

Forty samples were prepared as described above and were divided into groups. Five were used for the reference group, while the other 35 were heat treated at 800 °C to investigate the grain growth kinetics of the alpha phase (seven groups of five samples for each of the seven different annealing times: 10, 20, 30, 40, 60, 100, and 120 min).

Each group was placed in an oven at 800 °C for the desired annealing time, then removed from the oven and quenched in water at 20 °C. All samples were cooled uniformly by rapid quenching to prevent unintentional residual stresses, thus preventing excessive grain growth [22,23]. All samples were protected in an inert argon atmosphere to prevent any possible oxidation of the titanium surface, which could increase its brittleness.

After each different heat treatment, microhardness was studied to determine the annealing time that was associated with a recovery of the initial hardness of the material. The compressive stress caused by sandblasting treatment increases the hardness of the material, so when the compressive stress is eliminated as a result of the heat treatment, the original hardness is restored [24,25].

#### Microhardness Tests

Microhardness tests were performed with a Matsuzawa microhardness tester (Tokyo, Japan) using a diamond with a square base as a penetrator and applying a 1 kgf load for 15 s. Results were expressed as Vickers hardness values (HVN).

### 2.3. Metallographic Analysis

Samples were treated by metallographic polishing and chemical etching with a solution containing HF and HNO_3_, both at 15%. Metallographic analysis was conducted using an optical microscope (Olympus, Tokyo, Japan). Grain size was determined using image analysis techniques with OmniMet 3 (Buehler, Lake Bluff, IL, USA).

### 2.4. Implants

**Figure 2:**
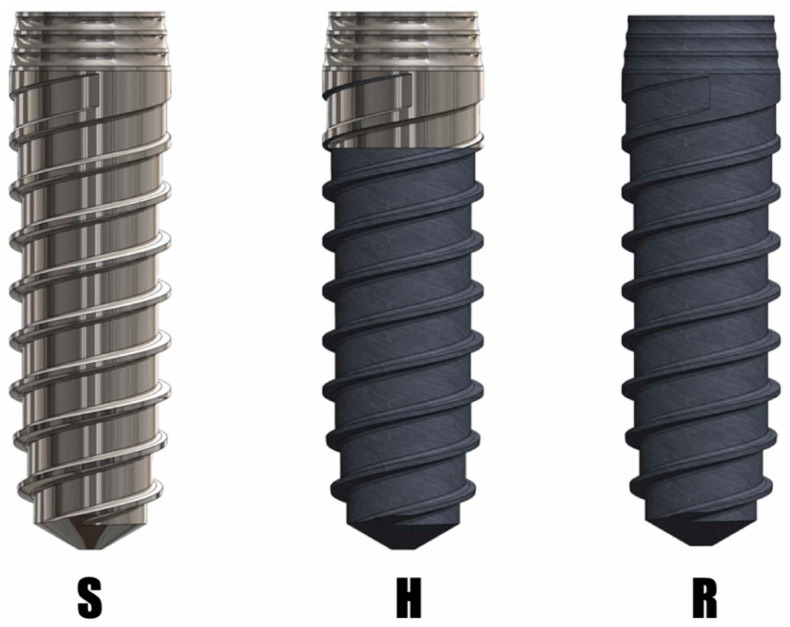
Implants in the study. S: machined surface. H: hybrid surface. R: rough surface (SLA).

Twenty hybrid implants, CpTi Grade III, provided by Klockner Dental Implants (Madrid, Spain), were analyzed. After heat treatment (800 °C) using an annealing time that could restore the original titanium hardness, five implants were used to assess the residual stress, and another five for corrosion resistance. The mechanical properties of these hybrid implants were compared with those of ten machined-surface implants and ten rough surface implants (SLA treatment) used in two previous studies by the same research team [14,16], with the latter serving as the control group (Figure 2).

### 2.5. Corrosion Resistance

A total of 30 samples, 10 samples per group, were used for the corrosion tests. The test area for each sample was 19.6 mm^2^. The electrolyte for all tests was Hank’s solution (ThermoFisher, Madrid, Spain) (Table 2), which is a saline fluid that closely captures the ion composition of the human serum environment [26,27,28].

**Figure 3:**
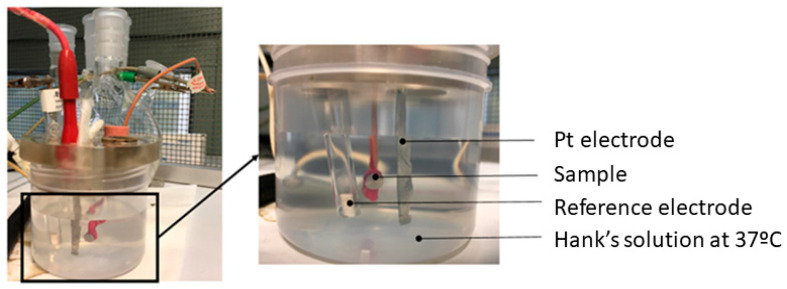
Corrosion resistance equipment.

The electrochemical cell used was a polypropylene (PP) container with a capacity of 185 mL and a methacrylate lid with 6 holes for the introduction of the sample, the reference electrode, and the counter electrode (Figure 3). The reference electrode was calomel (saturated KCl) for open circuit potential and potentiodynamic tests. The potential of this electrode is 0.241 V compared with the standard hydrogen electrode. All tests were performed at room temperature and in a Faraday box.

The calomel electrode and the sample were placed in the electrochemical cell to determine the corrosion potential results in an open circuit. Measurements were analyzed for 5 h, taking results every 10 s. The potential was accepted when the variation in the potential was lower than 2 mV for 30 min, in accordance with the ASTM E3-11 and ISO 10993-5 standards [29,30]. This test assesses which materials are more noble (higher potential), and thus less susceptible to corrosion. The data and the E-t curves were obtained using PowerSuite software (version 4.0, Oak Rideg, TN, USA) with the PowerCorr-Open circuit.

Potentiodynamic polarization curves were obtained for the 3 study groups, taking into account the ASTM G5 standard [31]. In this test, a variable electrical potential is imposed by the potentiostat between the calomel electrode and the sample, producing a current to flow between the sample and the counter electrode. The counter electrode used was platinum [28]. Initially, the system was allowed to stabilize by means of an open-circuit test for 1 h. After stabilization, this test was launched, performing a cyclic sweep from −0.8 mV to 1.7 mV at a speed of 2 mV/s. These parameters were registered into the PowerSuite software, and the PowerCorr-Cyclic Polarization function can be used to obtain the graphs. The results studied were:

- EOCP (mV)/Corrosion potential in open circuit
- icorr (μA/cm^2^)/corrosion current density.
- Ecorr (mV)/Corrosion potential: value at which the current density changes from cathodic to anodic.

The Ecorr and icorr parameters are obtained by extrapolating the Tafel slopes. The Tafel slopes are also used to obtain the Tafel coefficients: anodic (βa) and cathodic (βc). These coefficients represent the slopes of the anodic and cathodic branch, respectively, in accordance with the ASTM G102-89 standard [32].

Corrosion resistance of heat-treated hybrid implants was assessed by open circuit potential (EOCP), corrosion potentials (ECORR), and corrosion currents (ICORR) tests, according to previous studies [13].

### 2.6. Mechanical Properties

#### 2.6.1. Residual Stress

Residual stress was measured with a Bragg–Brentano diffractometer (D500, Siemens GmbH, Berlin, Germany), according to previous tests carried out by the same research team [16].

#### 2.6.2. Fatigue Behavior

Fatigue was assessed on 10 rough dental implants with a surface tension of −201 MPa (control group, SLA treatment), 10 hybrid dental implants with a residual stress difference between the smooth (−20.2 MPa) and rough (−201 MPa) portions, and 10 hybrid dental implants that were exposed to annealing heat treatment. The annealing heat treatment aimed to eliminate all internal energies, since the annealing temperature exceeds the recrystallization temperature of the tested titanium.

#### 2.6.3. Finite Element Analysis

A simplified model of the prosthesis-implant-bone system was created using Salome CAD software (Open Cascade SAS Company, Guyancourt, France) [33]. The implants were designed with a cylindrical body that was slightly enlarged in the upper region. All implants were assumed to have the same properties, design, and macrogeometry, except for residual stress (machined: −20.2 MPa, rough: −201 MPa, hybrid with heat treatment: −20.2 MPa).

**Figure 4:**
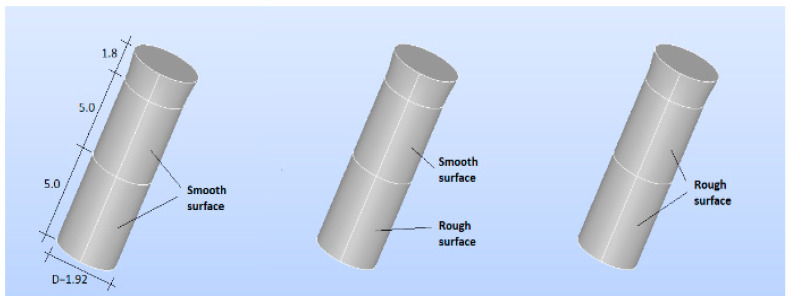
(**Left**): SS implant. (**Center**): SR implant. (**Right**): RR implant (dimensions in mm).

The three types of implants were then analyzed (Figure 4):

1. Smooth implant (SS): The entire implant was smooth.
2. Smooth-Rough implant (SR): The bottom part of the implant was rough, while the top part was smooth
3. Rough implant (RR): The entire implant was rough.

**Figure 5:**
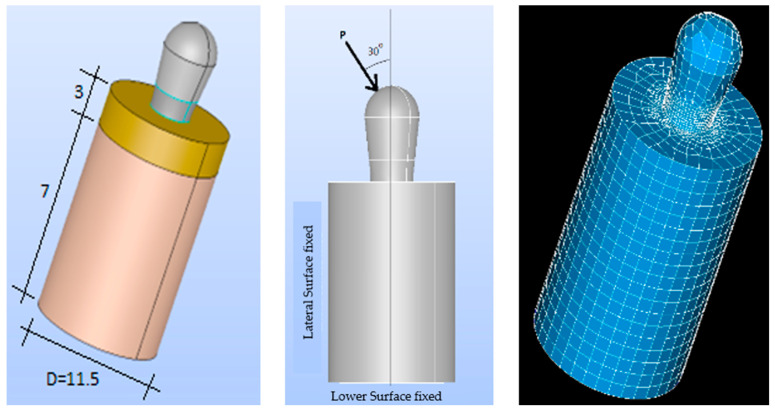
Upper-cortical model. (**Left**): implant model. (**Center**): loads. (**Right**): finite element mesh (56,227 elements and 109,328 nodes. Dimensions in mm.).

**Figure 6:**
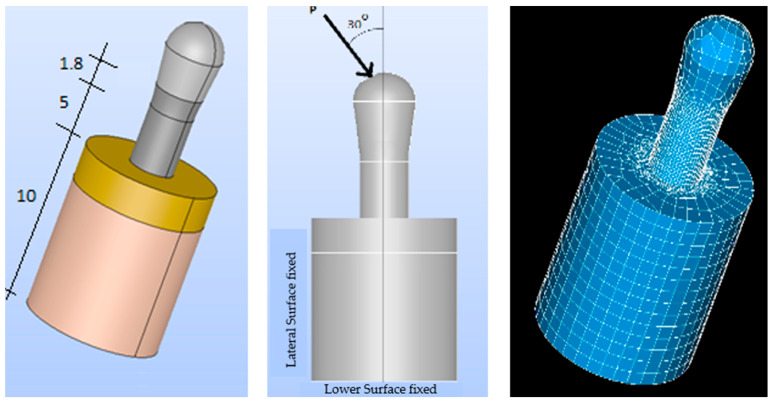
Lower-cortical model. (**Left**): implant model. (**Center**): loads. (**Right**): finite element mesh (79,234 elements and 101,452 nodes).

The surrounding bone was also modeled as a cylinder with two distinct regions to represent the cortical bone and the trabecular bone (Figure 5 and Figure 6). Two three-dimensional (3D) models were created, as they more accurately represent the implant and bone responses than two-dimensional (2D) models [34].

- The cortical bone (upper cortical): In the most coronal 5 mm, with a thickness of 5 mm (Figure 5).
- The trabecular bone (lower cortical): Spongy, represented in the most apical 5 mm (Figure 6).

A 3D model, preprocessing, and a finite element mesh were generated. The results were exported to Code-Aster (EDF France, Paris, France) for finite element analysis [35]. Prior to the numerical computation, a mesh-sensitive analysis was performed. As a result, the proposed model was meshed using classic quadratic tetrahedral elements with different refinements, with 1 mm as the element size for the bone and 0.2 mm for the implant components (Figure 5 and Figure 6).

A sensitivity analysis of the finite element mesh was performed before proceeding with the numerical calculations. As a result of this analysis, the proposed model was meshed by using classical quadratic tetrahedral elements with different refinements: a 1 mm element size for bones and a 0.2 mm element size for the components of the implant (see Figure 5 and Figure 6).

#### Mechanical Properties and Interface Conditions

All elements were considered isotropic, homogeneous, and linear elastic. Moreover, the bone-to-implant contact was assumed to be ideal for osseointegration (100%). Table 3 describes the mechanical properties of the model as reported in the literature [36,37].

#### Loading Conditions

The combined model (implant-bone) was inserted in all directions. Movement of the lateral and bottom surfaces was restricted to simulate the actual anchorage of the implant to the bone (Figure 5 and Figure 6). A compressive loading was applied to both models at an inclination of 30° with respect to the longitudinal axis of the implant. A compressive strength of 140 N was evenly distributed over the top of the implant.

## 3. Results

Microhardness values of the titanium disks are shown in Table 4, according to the different annealing times of the heat treatment. The microhardness of the rough areas (with compressive residual stresses due to grit blasting) was investigated.

The detailed results are shown in Table 4. The material demonstrated a microhardness of 115 HVN following 30 min of heat treatment at 800 °C, which was very close to that of titanium without residual stresses (110 HVN). Considering this, the 30 min annealing heat treatment was used in this study to eliminate the compressive residual stress of the hybrid dental implant, and was also used in all other tests. The fatigue and corrosion resistance analyses were performed with implants treated using this annealing regime. The decrease in microhardness values is due to grain growth. As the crystal surface area is larger, the movement of dislocations is favored, and hardness decreases.

### 3.1. Grain Growth

**Figure 7:**
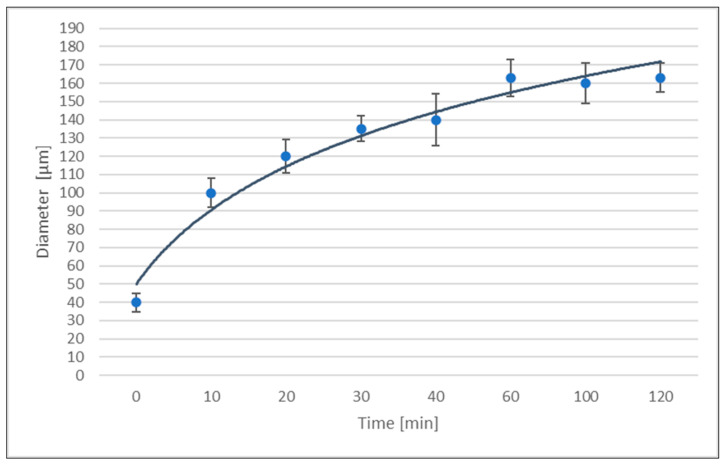
Mean grain size diameter as a function of annealing heat treatment time at 800 °C.

Grain size (mean diameter) as a function of time for the annealing heat treatment at 800 °C is shown in Figure 7. The initial grain diameter of the untreated implants was 40 µm.

Figure 7 shows that grain growth occurs very rapidly in the initial 10 to 20 min of the annealing heat treatment, and then tapers. This decrease can be attributed to the increase in grain size that decreases the grain boundary area per unit volume. This could indicate that the interfacial energy of the grain boundary per unit volume decreases and the driving force for grain growth is thus lower.

Since grain size was apparently the same in the longitudinal and transverse directions, new grain size measurements were performed only in the cross-section of the extruded bars. Texture is not likely to have a major effect, since the microstructure of the extruded samples showed complete recrystallization.

Grain growth kinetics follow the Hillert distribution, as the maximum radius is 1.8 times higher than the mean value of the radius. Therefore, a uniform growth occurs throughout the sample and size distribution follows an asymptotic stability typical of an equilibrium state [38]. Grain size data plotted as a function of log D versus log t yields a straight line. Hence, grain growth follows the general equation as follows [39,40]:

D – D_0_= Kt^2^

where D is the mean grain size, D_0_ is the initial grain size, K is a temperature-dependent constant, t is time, and *n* is the exponent obtained from the slope of the log D versus log t plot.

Moreover, if it is correct to assume that atomic diffusion across the grain boundary is a simple activated process, when *n* is independent of temperature, K can be calculated by [41] as follows:

$$K=K_{0}\exp-\frac{Ea}{Rt}$$

where *Ea* is the activation energy, *T* is the temperature in Kelvin, K_0_ is the pre-exponential factor rate constant, and *R* is the gas constant. This can explain how a Ln(D) versus 1/T plot produces a straight line. In that case, the slope should be −Ea/R.

Observed kinetic behaviors agreed with these assumptions. Linear equations with good correlation coefficients were obtained after plotting logarithms. The growth exponent *n* at 800 °C is shown in Table 4. However, these values were higher than those found for other metals and alloys [38,42,43]. Ideally, the growth exponent should be 0.5, but *n* is usually lower than 0.5 due to the role played by different grain growth parameters such as impurity-drag, free surface effect, texture, dislocation substructure, and heterogeneities [44,45]. In our case, the tested titanium studied showed a high degree of purity and very low dislocation density as a completely recrystallized metal. It is probably for this reason that the growth exponent is around 0.5 (Table 5) [46].

The activation energy for grain growth for the different heat treatment regimes was 100 kJ/mol for the alpha phase. Reinbach and Nowikow studied the effects of solute addition on the recrystallization of titanium. Among the added solutes (iron, aluminum, tantalum, tin, and chromium), chromium had the greatest effect in reducing the recrystallisation rate. This explains the low activation energies in commercially pure titanium. Reinbach and Nowikow found activation energies of 95 kJ/mol for alpha phase of alloy Ti-6Al-4V, 203 kJ/mol for Ti-6Al-5Zr-0.5Mo-0.25Si, and 211 kJ/mol for Ti-5.6Al-3.5Zr-1Nb-0.25Mo-0.3Si [42,47].

### 3.2. Corrosion Resistance

Table 6 and Table 7 show the mean values of EOCP, ECORR, and ICORR for machined (L), rough (R), untreated hybrid (H), and heat-treated hybrid (H + HT) implants. Values with an asterisk (*) represent statistically significant differences. It has been demonstrated in vitro that the large residual stress difference between the two surfaces of hybrid implants without heat treatment favors the appearance of corrosion pits at the interface, thus decreasing its corrosion resistance compared to single-surface implants (machined and rough) [14]. Our study showed that the effects significantly improve following residual stress homogenization.

### 3.3. Residual Stress

The compressive residual stress of heat-treated hybrid implants was similar to that of machined implants (20.2 Mpa) (Table 8). After heat treatment, stress was homogenized throughout the surface. The stress was still significantly higher among rough implants (202 MPa) (*p* ˂ 0.05, *t*-student).

### 3.4. Fatigue Behavior

**Figure 8:**
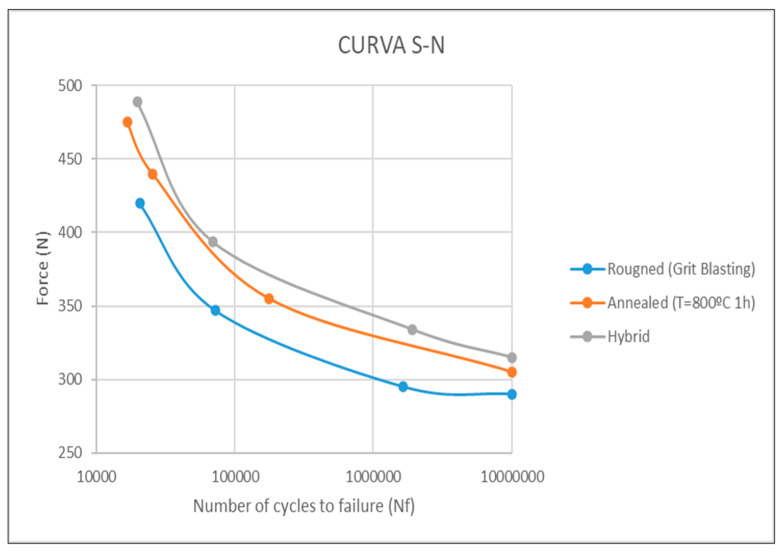
S-N curve of rough dental implants produced by grit blasting treatment, hybrid dental implants, and hybrid dental implants with annealing heat treatment.

Fatigue behavior is depicted in the S-N Curve in Figure 8. As observed, residual stress homogenization of the hybrid dental implants after the annealing heat treatment increased fatigue compared to the hybrid implants with residual stress differences. Prior to treatment, they had the worst values compared to implants with machined and rough surfaces. All cracks nucleated at the interface between the two surfaces, which represents the area of greatest stress concentration [16]. This change in fatigue behavior demonstrates that the stress difference is the main cause of the decrease in fatigue life.

Moreover, dental implants with compressive residual stress throughout the dental implant, which is the case of sandblasted dental implants, still have the best fatigue life of those studied, since compressive stress prevents fatigue crack generation. Grain growth can contribute to a decrease in fatigue resistance since it slightly decreases mechanical strength. However, the variations are not very important, and the heat treatment does not cause a significant decrease due to the increase in grain size.

### 3.5. Finite Element Analysis

**Figure 9:**
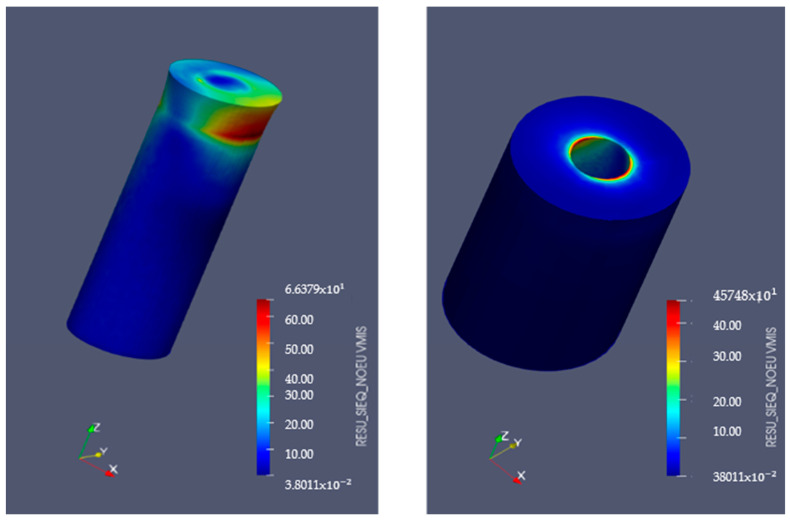
Implant Von Mises stresses (MPa) in upper-cortical model. Left: implant. Right: bone.

**Figure 10:**
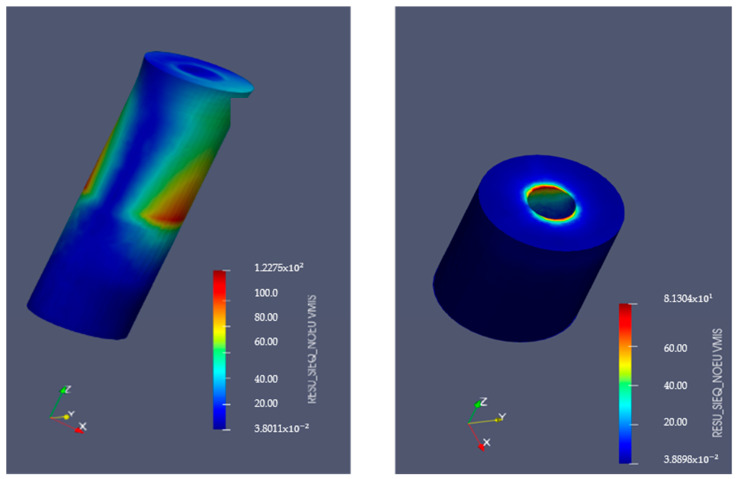
Implant Von Mises stresses (MPa) in lower-cortical model. Left: implant. Right: bone.

**Figure 11:**
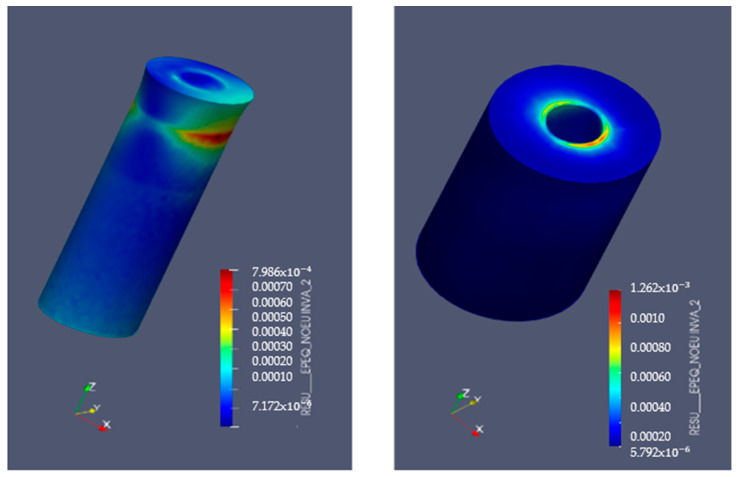
Implant microstrains in upper-cortical model. Left: implant. Right: bone.

**Figure 12:**
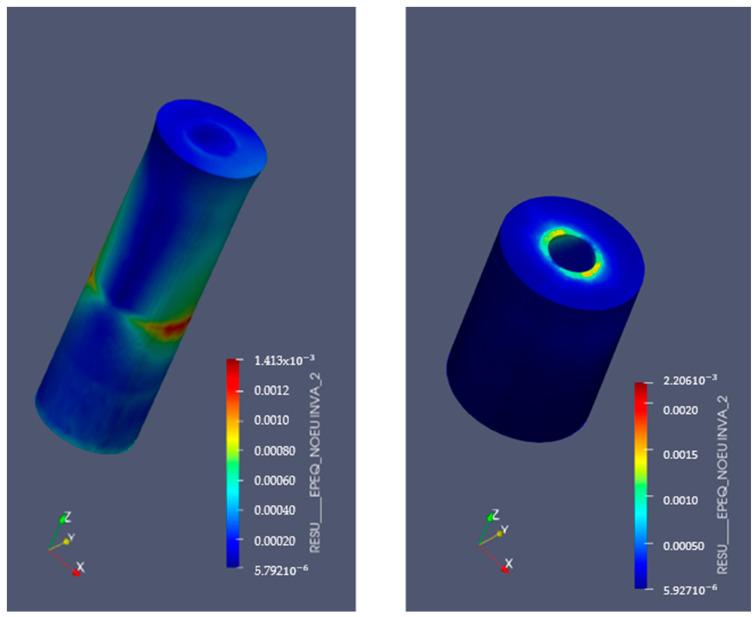
Implant microstrains in lower-cortical model. Left: implant. Right: bone.

Figure 9 and Figure 10 display the von Mises stresses obtained at both the upper cortical and lower cortical models, respectively. Figure 11 and Figure 12 show the von Mises stresses (MPa) and microstrains (mm/mm) of the implant and at the bone.

Von Mises stresses increased considerably in the heat-treated hybrid implants and trabecular bone, as represented in the lower cortical models. This stress increase may indicate a better distribution of forces at the bone-implant interface, favoring the maintenance of its equilibrium and stimulating its continuous adaptation to loading. Stress distribution analysis in hybrid implants with treated machined coronal portions and in single-surface machined implants in the upper cortical bone showed that the former has slightly lower stresses when compared to implants with a fully smooth surface, a consequence of the elimination of the residual stress inherent to the material by the machining process.

**Figure 13:**
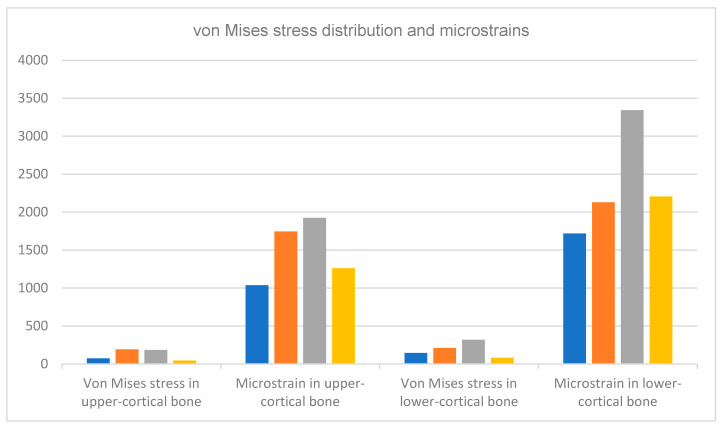
Comparison of von Mises stress values (MPa) and resulting microstrains (mm) in the upper- cortical and lower-cortical bones of the four implant groups (implant with machined surface, rough SLA, hybrid without heat treatment, and hybrid with heat treatment) using finite element analysis [16].

Consistent with those stress distributions, microstrains increased significantly both in treated hybrid implants and in the bone of the lower cortical models, and were slightly lower in the upper cortical bone when compared to fully machined surfaces (Table 9 and Figure 13).

## 4. Discussion

The main objective of this in vitro study was to improve the mechanical behavior of the hybrid implants and their biocompatibility by reducing the risk of corrosion through residual stress homogenization.

Residual stresses are intrinsic stresses of a material and exist even when no load is applied. In fact, intrinsic stresses should be considered as additional to loading stresses. Failure to do so could cause the material to fail earlier than expected. Stresses can also be introduced during the manufacturing process and when certain treatments, e.g., shot blasting, are applied. Compressive stresses (e.g., in rough surfaces prepared by shot blasting) significantly improve fatigue behavior, as they counteract crack nucleation, thereby partially neutralizing external tensile stresses [48,49]. Additionally, blasting alumina grit creates microcavities on the implant surfaces that shift crack initiation from the surface to 1–2 µm away from the surface.

At the interface of the two surfaces making up a hybrid implant, the residual stress is significantly different (−20.2 MPa in the machined surface versus −202 MPa in the rough surface). This causes non-homogeneous local plastic deformations that worsen the implant’s mechanical behavior, fatigue behavior, and stress distribution, as demonstrated in our previous study [16]. Corrosion potential also increases in hybrid implants, thus generating corrosion pits at the interface of the two surfaces that concentrate the stress [14]. Consequently, corrosion fatigue occurs, with the chemical action of corrosion converging with the mechanical action of cyclic loading.

The mechanical behavior of implants is dependent on its loads (e.g., axiality, cycles, and type), geometry and design, surface treatments (residual stresses and stress concentrators), and microstructure. Commercially pure titanium was used in our in vitro study. It has only one allotropic phase (the alpha phase), so heat treatment only changes grain refinement. Microstructure changes depend on the amounts of interstitial elements (in our case, it had high purity), heat treatment temperature, annealing time, and quenching rate. The heat treatment temperature should be set so as to ensure homogenization of the residual stresses, thereby improving the implant’s corrosion and fatigue resistance, without affecting its mechanical properties [50]. Other in vitro studies, such as those by Li et al., have demonstrated optimal results when exposing CpTi to temperatures in the range of 690–790 °C [51]. Vematsu et al. treated grade 2 titanium at a temperature of 1000 °C, higher than the β-transus. Although the hardness did improve, fatigue behavior worsened compared to the control samples [52].

In this in vitro experimental study, the annealing heat treatment homogenized the residual stresses at the interface, especially where the different surfaces converged. The heat treatment also improved their fatigue behavior and corrosion resistance when compared to untreated surfaces. A correlation was found between the annealing temperature, changes in the microstructure of the material, and its physical-chemical characteristics. An annealing temperature of 800 °C improved residual stress homogenization and an annealing time of 30 min provided the best fatigue behavior. Complete recrystallization was achieved at 800 °C, with equiaxed α grains and without excessive grain growth. When grain growth was considered normal, without recrystallizations, the driving force was the interfacial energy of the grain boundary [53]. When a temperature of 800 °C was used and maintained for a sufficiently long period (30 min), growth occurred by diffusion. Initially, growth occurred very rapidly (initial 10–20 min), and then slowed down as grain size increased, decreasing the number of grains per unit volume. Grain growth demonstrated a growth exponent *n* of 0.5 and reached a mean grain diameter of 160 µm, with no abnormal grain growth (which would have led to worse mechanical behavior). Values were homogenized in heat-treated hybrid implants, with a compressive residual stress of 20 MPa throughout the surface. Heat treatment of commercially pure titanium affects only grain structure, and the mechanical properties are not greatly modified, but corrosion resistance and residual stress homogenization is obtained, which, in turn, increases fatigue performance, and, ultimately, lifespan. When hybrid implants have not been heat treated, heterogeneous residual stresses between two heterogeneous surfaces can affect the implants’ mechanical properties.

Fatigue behavior was analyzed using traditional fatigue criteria and finite element analysis. The annealing heat treatment improved fatigue of annealed hybrid dental implants when compared to untreated hybrid implants. Annealing eliminates the residual stress differences that promote crack formation. For the rough dental implants, with a homogeneous stress of −201 MPa, a compressive stress state that hindered crack nucleation was generated, leading to the best fatigue behavior. Finite element analysis showed that von Mises stress distribution and microstrains significantly increased in both the lower-cortical bone and the heat-treated hybrid implants. The results obtained for untreated implants showed that the interface between the two surfaces would be the most likely area to fail and the coronal portion of the peri-implant bone would be at the greatest risk of resorption, since von Mises stress distribution was poor. Such changes in stress distribution improved bone adaptation to loading [54] and confirmed that implant surface conditions affect loading. It would be beneficial to carry out clinical studies to determine how changes in stress distribution affect the apposition or resorption of peri-implant bone tissue.

The annealing heat treatment also increased the implants’ corrosion resistance. The convergence of different residual stresses favors electrochemical pitting corrosion, especially at the interfaces of the rough and smooth parts, as they act as the anode and cathode, respectively. In this study, the annealing treatment improved corrosion resistance even in implants with machined surfaces only. This may be because heat treatment also eliminates residual stresses caused by dental implant machining. The grain growth increase caused by the 30 min heat treatment also contributed to the improved corrosion resistance. Grain boundaries are susceptible to pile formation since they separate and join grains with different orientations, thus favoring electrolytic attack. Fewer grain boundaries, created by grain growth, produce fewer pitting initiation points, and a higher current intensity is required for the attack [55,56].

There is, therefore, a relationship between microstructure, mechanical properties, and corrosion resistance after applying an annealing heat treatment. This relationship is established for any of the allotropic phases of titanium and its alloys (α, α + β and β). Ibrahim et al. [57] performed an annealing treatment to the Low-Cost alloy β Titanium. At a temperature of 650 °C, mechanical properties (hardness and tensile strength) were improved due to the obtained microstructure, conformed with equiaxed β grains and primary α-phase in 15% of the volume. By applying an annealing temperature of 750 °C, corrosion resistance was enhanced, thanks to the 5% reduction in the α phase, which was also anchored to grain limits. Likewise, Lavrys et al. [58] applied an annealing treatment to AMTi-6Al-4V (α + β). At a temperature of 800 °C, residual stresses were relieved, and corrosion resistance was improved by modifying their phase microstructure α’ metastable martensite, releasing the β phase. However, increasing the annealing temperature to 850 °C doubled the grain size, worsening the corrosion resistance compared to the first treatment.

Thus, very long annealing heat treatment or treatment at very high temperatures is not advisable, since grain growth increases exponentially and causes a very significant decrease in the elastic limit, maximum resistance, and hardness, as indicated by the Hall-Petch law.

Future clinical and in vivo studies are necessary to verify our results. This is especially important since some conditions are difficult to reproduce in in vitro studies. Various factors, such as the presence of intraoral fluid [59], bone loss with thread exposures, splinting implant restorations [60], and occlusal conditions, may influence the results.

## 5. Conclusions

Considering the findings of this in vitro study, the null hypothesis must be rejected. Thirty minutes of annealing heat treatment at 800 °C homogenized the residual stress differences between the two coexisting surfaces of hybrid implants. Residual stress homogenization improved the implants’ mechanical properties, raising fatigue behavior and von Mises stress distribution in the trabecular bone, confirming the influence of residual stresses. The corrosion resistance of heat-treated hybrid implants was also significantly improved. However, the heat treatment was not able to improve the mechanical properties of rough implants. It is again confirmed that rough implants have the best fatigue performance.

## Figures and Tables

**Table 1:** Chemical impurities (weight percent).

Nitrogen	Carbon	Hydrogen	Iron	Oxygen	Titanium
0.05	0.10	0.12	0.30	0.35	Balance

**Table 2:** Chemical composition of Hank’s solution.

Chemical Product	Composition (mM)
K_2_HPO_4_	0.44
KCl	5.4
CaCl_2_	1.3
Na_2_HPO_4_	0.25
NaCl	137
NaHCO_3_	4.2
MgSO_4_	1.0
C_6_H_12_O_6_	5.5

**Table 3:** Mechanical properties of the model.

Material	Young’s Modulus (MPa)	Poisson’s Ratio
Implant	110,000	0.32
Cortical bone	19,400	0.30
Trabecular bone	5600	0.28
Loading device	200,000	0.30

**Table 4:** Sample microhardness (Vickers hardness number) according to the different annealing time at 800 °C.

Time	HVN
0	234 ± 13
10	159 ± 7
20	120 ± 6
30	115 ± 7
40	110 ± 9
60	107 ± 7
100	104 ± 3
120	102 ± 4

**Table 5:** The K and n values for different treatment temperatures.

Temperature (°C)	log K (Temperature-Dependent Constant)	N (Growth Exponent)
800	0.98	0.50

**Table 6:** Open circuit potential (E_OCP_) for each type of dental implant.

Dental Implants	E_OCP_ (mV)
L	−200.9 ± 13.3
R	−192.2 ± 0.10 *
H	−186.4 ± 4.9 **
H + HT	−199.8 ± 2.3

* and ** indicate statistically significant differences between the three surfaces studied in each roughness parameter (*p* < 0.05) obtained by ANOVA. L: smooth implants. R: rough dental implant. H: hybrid surface implant. HT: Heat-treatment hybrid surface implants.

**Table 7:** Current density (J_CORR_) and corrosion potential (E_CORR_) of different dental implants.

Dental Implant	j_CORR_ (uA/cm^2^)	E_CORR_ (mV)
L	0.014 ± 0.055	−280 ± 53
R	0.019 ± 0.019	−273 ± 34
H	0.069 ± 0.015 *	−223 ± 50 *
H + HT	0.016 ± 0.034	−278 ± 24

* Symbols indicate statistically significant differences between the three surfaces studied in each roughness parameter (*p* < 0.05). L. obtained by ANOVA. L: smooth implants. R: rough dental implant. H: hybrid surface implant. HT: Heat-treatment hybrid surface implants.

**Table 8:** Residual stresses of the different implants.

Dental Implant	Residual Stress (MPa)
Machined implant	20.2 (5.3)
Rough implant	202.2 (11.2) *
Hybrid implant with annealing heat treatment	20.2 (5.3)

* Statistically significant.

**Table 9:** Maximum von Mises stresses (MPa) and microstrains (mm/mm) of the implant and the bone.

		Implant		Bone	
Model		Svon	µstrains	Svon	µstrains
Upper Cortical	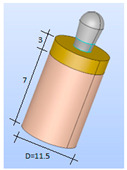	66.3	0.798 × 10^−3^	45.7	1.262 × 10^−3^
Lower Cortical	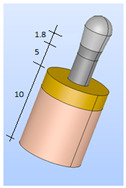	122.7	1.413 × 10^−3^	81.3	2.206 × 10^−3^

## Data Availability

The authors can provide details of the research requesting by letter and commenting on their needs.
